# Mesoscopic Conductance Fluctuations in 2D HgTe Semimetal

**DOI:** 10.3390/nano13212882

**Published:** 2023-10-31

**Authors:** Daniiar Khudaiberdiev, Ze Don Kvon, Matvey V. Entin, Dmitriy A. Kozlov, Nikolay N. Mikhailov, Maxim Ryzhkov

**Affiliations:** 1 Institute of Solid State Physics, Vienna University of Technology, 1040 Vienna, Austria; daniiar.khudaiberdiev@tuwien.ac.at (D.K.);; 2 Rzhanov Institute of Semiconductor Physics, Novosibirsk 630090, Russia; 3 Novosibirsk State University, Novosibirsk 630090, Russia; 4 Experimental and Applied Physics, University of Regensburg, D-93040 Regensburg, Germany

**Keywords:** quantum wells, universal conductance fluctuations, two-dimensional semimetal

## Abstract

Mesoscopic conductance fluctuations were discovered in a weak localization regime of a strongly disordered two-dimensional HgTe-based semimetal. These fluctuations exist in macroscopic samples with characteristic sizes of 100 μm and exhibit anomalous dependences on the gate voltage, magnetic field, and temperature. They are absent in the regime of electron metal (at positive gate voltages) and strongly depend on the level of disorder in the system. All the experimental facts lead us to the conclusion that the origin of the fluctuations is a special collective state in which the current is conducted through the percolation network of electron resistances. We suppose that the network is formed by fluctuation potential whose amplitude is higher than the Fermi level of electrons due to their very low density.

## 1. Introduction

Universal conductance fluctuations (UCF) are one of the most fundamental phenomena representing the quantum nature of the electron. Their discovery has led to the birth of the Mesoscopics [1,2,3,4,5]. One of their greatest properties is that their amplitude in the regime of weak localization is close to the quantum of conductivity ($<{\Delta G}^{2}{>}^{1/2}\approx e^{2}/h$) in a sample with the characteristic length $L<L_{T}$. At the finite temperature $<{\Delta G}^{2}{>}^{1/2}\approx \left(e^{2}/h\right)L_{T}/L$, where $L_{T}={\left(D\hbar /T\right)}^{1/2}$ is the coherence length and *D* is the diffusion coefficient. In most cases, $L_{T}=\left(0.1-1\right)$ μm at T≤1 K, and thus the UCF usually require metallic conductivity and microscopic sizes of samples to be observed. This means that any conductor of smaller micron sizes with metallic conductivity should demonstrate UCF. At the present time, there are a lot of papers concerning different properties of the UCF in ordinary mesoscopic metals, semiconductors, superconductors and hybrid mesostructures on the basis of them [6,7,8,9,10,11,12,13,14,15,16,17,18,19,20], in graphene-like structures [21,22,23,24,25,26,27,28,29,30], and in mesoscopic topological systems [31,32,33,34,35,36,37,38,39,40,41].

In this paper, we report the observation of the mesoscopic conductance fluctuations in a strongly disordered semimetal implemented in the samples of macroscopical sizes (L≥100 μm) with significantly different behavior compared to the UCF. It is suggested that the fluctuations are caused by the collective state in the unbalanced semimetal (relation of the hole and electron concentrations $P_{s}/N_{s}\geq 10^{2}$) where the current passes through the electron percolation network created by screening the fluctuation potential by heavy holes.

## 2. Samples

**Figure 1:**
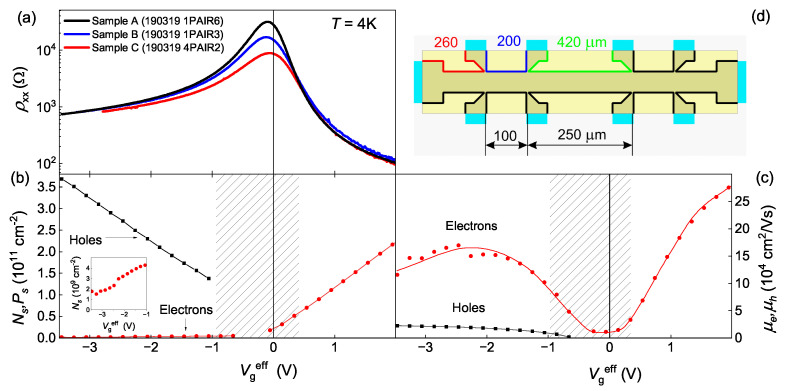
(**a**) Gate voltage dependences of resistivity at T=4 K in samples A, B, and C. $V_{g}$ is shifted for $V_{CNP}$—the position of the charge neutrality point (CNP) is different for each sample. (**b**) Gate voltage dependence of the electrons ($N_{s}$) and holes ($P_{s}$) density in sample A derived from the fitting of magnetotransport by the Drude model. (**c**) Gate voltage dependence of the mobilities of electrons $\mu_{e}$ and holes $\mu_{h}$ in sample A derived from the fitting of magnetotransport by the Drude model. (**d**) The scheme of the ten contact Hall bar. The width of the channel is 50 μm, the distances between contacts are 100 and 250 μm. The area covered by the gate is represented by the golden rectangle.

The studied samples are the field-effect transistors (their topology and distances are depicted in Figure 1d) based on HgTe (013) quantum wells (QW) 14 nm width with TiAu gates. Let us notice that this two-dimensional semimetal has an overlap of the conduction and valence bands of the same order as in the well-studied 18–22 nm HgTe QW, but with lower mobilities of electrons and holes. Its detailed study is given in [42,43]. The measurements were carried out in the temperature range from 0.08 K–4 K and in magnetic fields up to 2 T with the standard lock-in technique in the frequencies from 0.3 to 12 Hz and the current 0.1–10 nA depending on the state of the system.

Figure 1a demonstrates gate voltage dependences of the studied samples’ resistivity $\rho_{xx}\left(V_{g}^{eff}=V_{g}-V_{CNP}\right)$, where $V_{g}^{eff}$ is counted from the charge neutrality point (CNP), at the temperature T=4 K. The CNP gate voltages $V_{CNP}$ are −0.5 V, −0.2 V and −1.2 V for samples A, B, and C, respectively. As one can see, the curves have maxima at $V_{g}^{eff}\approx -0.1$ V, with resistivity equal 32, 17 and 9 kΩ/☐. Since the samples were fabricated from the same wafer, and they have different maximum resistivity; we can further use them to analyze the impact of disorder presented in the system. It is worth noting that at $V_{g}^{eff}<-2.7$ V, $\rho_{xx}\leq 1\;$kΩ/☐, which means it precisely corresponds to the weak localization regime ($k_{F}l>$ 20, $k_{F}$—the wave vector of charge carriers, *l*—their momentum relaxation length).

The concentrations and mobilities of the electrons and holes in the sample A are shown in Figure 1b and Figure 1c, respectively. They were determined from magnetotransport measurements (as in [42]). At the gate voltages −3.5 V $\leq V_{g}^{eff}\leq$−1 V the studied semimetal is strongly unbalanced with the holes concentration $P_{s}$ being one to two orders higher than the electron concentration $N_{s}$. At the same time, the relation of the mobilities is the opposite (Figure 1c). Thus, at the considered gate voltages region, the system is a strongly unbalanced semimetal with metallic conductivity so that the contribution to the conductivity from a very small amount of light electrons ($m_{e}\approx 0.03m_{0}$) is comparable to the one from a high amount of heavy holes ($m_{h}\approx 0.25m_{0}$) [44]. Let us also notice that the semimetallic band structure (studied in Reference [44]) has a small band overlap Δ of approximately 5 meV. This allows us to reach such an unbalanced semimetallic state by applying the gate voltage.

## 3. Results

In this section, we present the discovered mesoscopic fluctuations and their properties. We begin with the conductance fluctuations in the gate voltage dependences at different magnetic fields, temperatures, and different parts of the sample. Further, we show the fluctuations in the magnetic field dependences, and finally, we compare the results from different samples.

**Figure 2:**
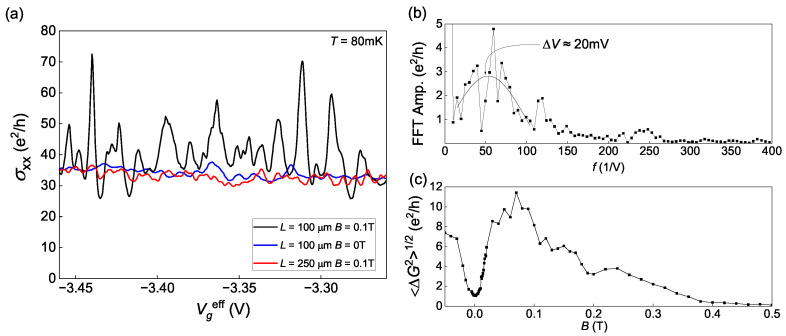
(**a**) Gate voltage dependence of conductivity. (**b**) The Fourier spectrum of the conductance. (**c**) Magnetic field dependence of the average fluctuations amplitude.

The main result is presented in Figure 2a. It shows gate voltage dependences of the conductivity $\sigma_{xx}$ measured on a short part of the Hall bar (with 100 μm distance between potentiometric contacts) in a short range of gate voltages from −3.46 V to −3.26 V at the temperature of 0.08 K in the absence of magnetic field (blue curve) and in magnetic field B= 0.1 T (black curve). It has also been measured on a long part of the sample with 250 μm between contacts (red curve). Conductivity $\sigma_{xx}$ is calculated as $\rho_{xx}^{-1}$ since $\rho_{xy}$ is relatively small at given magnetic fields.

One can clearly see the fluctuations with the average period of 20 mV (see the Fourier spectrum of $\sigma_{xx}\left(V_{g}\right)$ at Figure 2b) and with the amplitude about $e^{2}/h$, which increases by an order after applying the magnetic field. Figure 2c shows how the average fluctuations amplitude depends on the magnetic field: it increases by an order in a range from 0 to 0.03 T, reaches a maximum at 0.03 T <B<0.2 T and then falls to zero when the magnetic field reaches 0.5 T. One may also see that the fluctuations of $\sigma_{xx}$ in the field of 0.1 T measured on the long part of the sample are much weaker. That means that the observed fluctuations depend on the size of the conductor, and, in this aspect, their behavior is similar to the UCF; thus, they could be called mesoscopic. However, all the other features, including anomalously large sizes of the conductor, a sharp increase with the application of the magnetic field, and their shutdown after 0.5 T, distinguish them from the UCF.

**Figure 3:**
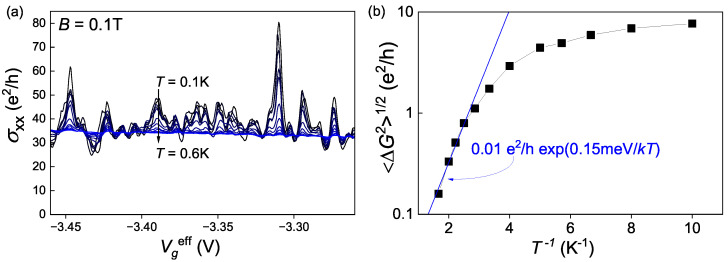
(**a**) Conductance fluctuations at different temperatures. The colors of the lines change depending on the temperature from black (at *T* = 0.1 K) to blue (at *T* = 0.6 K). (**b**) Temperature dependence of the conductance fluctuations mean amplitude.

Another important feature of the fluctuations is their temperature dependence. In fact, we were quite surprised to observe them because the measurements at relatively high temperatures (from 1 K to 4 K) showed no signs of such fluctuations. We expected that, at lower temperatures, we would observe weak localization effects, but instead, we discovered huge mesoscopic fluctuations. Figure 3a shows $\sigma_{xx}\left(V_{g}^{eff}\right)$ dependences at different temperatures in range 0.1 K ≤T≤ 0.6 K, and Figure 3b shows the temperature dependence of the fluctuations amplitude. The amplitude of the fluctuations sharply decreases with the increase in the temperature. In the temperature range from 0.6 K to 0.3 K, the temperature dependence is exponential with the activation energy of 0.15 meV, but then, at lower temperatures, it starts saturating. Saturation of this kind could indicate the existence of a distinct fluctuating regime to which the system switches at low temperatures. On the other hand, the activation behavior at higher temperatures may indicate a process that suppresses the fluctuations. Further, we will discuss the temperature dependence in Section 4.2.

**Figure 4:**
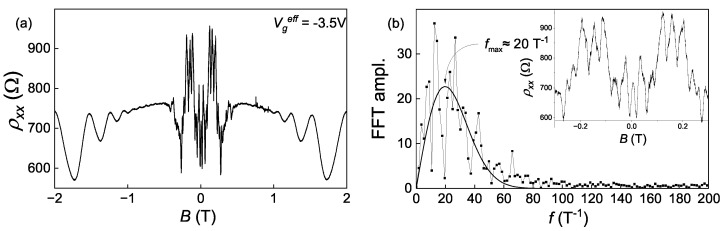
(**a**) Magnetic field dependence of resistivity at $V_{g}^{eff}$ = −3.5 V. (**b**) The Fourier spectrum of low-field oscillations from (**a**).

These fluctuations are not only observed in the gate voltage dependences of conductivity but also in their magnetic field dependences. The typical magnetic field dependence of $\rho_{xx}$ at fixed gate voltage is presented in Figure 4a. It demonstrates quasi-periodical oscillations of $\rho_{xx}$ in the fields |B|<0.5 T. At the fields higher than 0.5 T they are already absent (what agrees with Figure 2c), and in the fields around 1 T Shubnikov–de Haas oscillations of 2D holes take their place. The transition of the system to the Shubnikov–de Haas regime, where the Landau level quantization becomes important, could be a reason for the mesoscopic fluctuation shutdown. It is worth noting that these fluctuations exhibit a perfect symmetry under the sign change of the magnetic field that is similar to the UCF.

The Fourier spectrum of the low-field quasi-periodical oscillations is shown in Figure 4b. The spectrum lacks a distinct frequency, implying that the oscillations are not perfectly periodic but rather fluctuating. Nevertheless, we can identify a characteristic frequency of $f_{max}\approx 20\;T^{-1}$ and thus a period of 50 mT. When considering these fluctuations as Aharonov–Bohm h/2e oscillations for future estimations, we can derive a characteristic area of the resonator S≈0.04 μm${}^{2}$ (or a characteristic size a≈0.2 μm).

**Figure 5:**
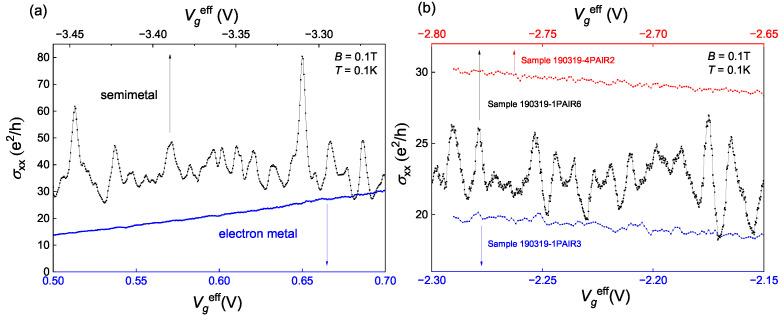
(**a**) Gate voltage dependences of conductance in semimetal (−3.46 V$<V_{g}^{eff}<$−3.26 V) and electron metal (0.5 V $<V_{g}^{eff}<$ 0.7 V) regimes. (**b**) Gate voltage dependences of conductance of samples with different disorders.

To determine the nature of the observed mesoscopic fluctuations, it is essential to note that they occur exclusively in the semimetallic state (at negative gate voltages) but not in the electron metal state (at positive gate voltages). Figure 5a presents $\sigma_{xx}\left(V_{g}^{eff}\right)$ in both semimetal and electron metal gate voltage regions at B=0.1 T and T=0.1 K. It is clear that the first one is fluctuating and the second one is smooth. Therefore, we conclude that the origin of the found fluctuations must be connected to the semimetallic nature of the studied state.

All the previous results were presented from sample A, and now we compare the results across all the samples. Gate voltage dependences $\sigma_{xx}\left(V_{g}^{eff}\right)$ of the three samples with different levels of disorder are presented in Figure 5b. The samples A, B, and C have different maximum resistance (see Figure 1a) and thus, different levels of disorder. Sample A, being the most disordered one, has the highest fluctuations amplitude, and sample C, being the purest one, has the weakest fluctuations. Additionally, apart from the fluctuations in the weak localization regime, conductivity does not depend on the disorder as much.

**Figure 6:**
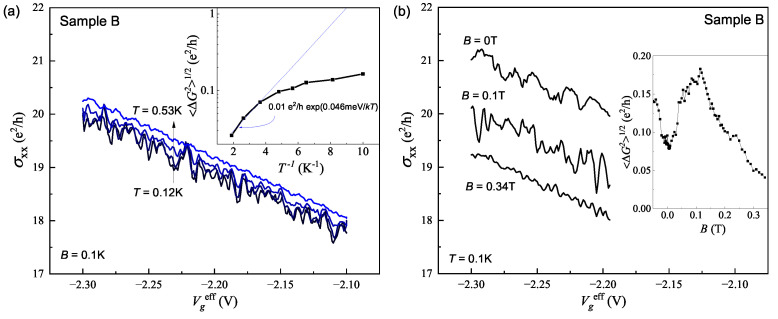
Sample B. (**a**) Gate voltage dependences of conductance at different temperatures. The inset shows the inverse temperature dependence of the fluctuations’ amplitude. (**b**) Gate voltage dependences of conductance at different magnetic fields. The inset shows the magnetic field dependence of the fluctuations’ amplitude.

Since sample B also exhibits significant fluctuations, let us present the data and compare them to sample A. The gate voltage dependences of the conductivity at different temperatures and magnetic fields are displayed in Figure 6a and Figure 6b, respectively. The insets in the graphs depict the amplitude of the fluctuations as a function of inverse temperature and magnetic field. Despite the substantial difference in the amplitude of the fluctuations between samples A and B, the temperature and magnetic field dependences appear quite similar. As in Figure 2c, we observe that in a zero magnetic field, there are some fluctuations. With a small magnetic field of approximately B≈40 mT, the amplitude significantly increases, reaching its maximum at B≈0.11 T. As in Figure 3b, at low temperatures, the amplitude tends to saturation, and at higher temperatures, there is an exponential decrease. Thus, we observe mesoscopic fluctuations in macroscopic samples of different disorder levels. The amplitudes of the fluctuations in these samples are different, but they exhibit similar magnetic field and temperature dependences.

## 4. Discussion

### 4.1. The Nature of Fluctuations

Despite some similarities to the UCF, the discovered fluctuations are vastly different. The largest difference is that they occur in macroscopical samples when the characteristic sizes of the sample are much larger than the coherence length ($L\gg L_{T}$) and only in a highly unbalanced semimetallic state as the hole density is much higher ($P_{s}/N_{s}\approx 10^{2}$) than the electron density. Their amplitude strongly depends on the magnetic field, and in the range of magnetic fields between 0.03 T and 0.15 T, it exceeds $e^{2}/h$ by an order. Their temperature dependence is abnormal (strongly exponential rather than weak linear), and their existence crucially depends on the disorder level.

It seems like the nature of the observed fluctuations, as in the case of the UCF, is also the quantum interference. The difference is that in our case, despite the fact that the sample is large, there is no averaging of the fluctuations. This is similar to the case of interfering squared network of metal conductors [45,46,47].

**Figure 7:**
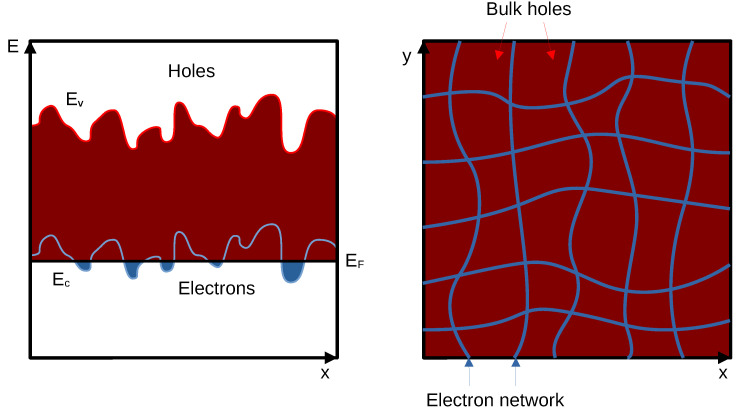
Schematic band diagram and the map of current channels in the presence of fluctuations.

This analogy with the squared electron network leads us to a possible explanation of the observed fluctuations. We suppose that such a small concentration of electrons (see Figure 1b) with a high level of disorder in the system can result in the formation of the electron network. The qualitative impurity potential fluctuations representation and current channels map of highly unbalanced semimetal including fluctuational potential with amplitude less than the hole’s Fermi level but higher than the electron Fermi level are shown in Figure 7.

One can see that while for holes we have small relative density fluctuations (Δ*P_s_* < *P_s_*), for electrons, there are even areas with zero density and areas with *N_s_* > 0, i.e., the actual percolation network of electron resistances. The only difference from an ordinary percolation network is that there are hole “lakes” between electron “rivers” rather than “mountains”.

The requirements for the mesoscopic fluctuations to appear in such a percolation network are significantly simpler than in a usual 2D electron system. The decoherence length of an electron needs to be comparable not to the length of the sample but rather to the characteristic size of the network’s element. This size is estimated from the period of magnetoresistance oscillations (see Figure 4) as a≈200 nm, which is by three orders of magnitude smaller than the sample’s characteristic size. These estimations allow us to propose that in strongly unbalanced and disordered 2D semimetal in HgTe QW forms a collective state of an electronic percolation network inside of the hole liquid.

### 4.2. Qualitative Model of the Transport

Given the complexity of the system under study, which includes impurities, heavy holes, light electrons, and potentially even 2D topological insulator states, we do not attempt to propose a comprehensive theory for the observed fluctuations. In this subsection, we present a qualitative model that does not perfectly describe the experiment but provides some explanations for the experimental observations and ideas for future microscopic theories. Here are the main assumptions of our model:

- Firstly, as has already been discussed, we assume that in the presence of impurities and heavy holes, the electron subsystem forms a percolation network. It could be similar to the squared network of electrons from References [45,46,47], but much less ordered. The fact that the formation of the percolation network crucially depends on a random potential of impurities explains the difference in the fluctuations amplitudes of samples A, B, and C with different levels of disorder. The question of how such a network actually forms we will leave to future theoretical studies;
- Secondly, we make the assumption that at low temperatures, the electron and hole subsystems are independent, while at higher temperatures, they exhibit strong interactions. Indeed, the electron-hole scattering is present in HgTe semimetals, resulting in a resistivity increase proportional to $T^{2}$ [48]. It also appears that direct transitions of an electron between the conduction and valence bands, which could even be present at zero temperature, are suppressed due to the significant distance between the electron and hole subbands in the momentum space. Conversely, electron-hole scattering occurs as an electron and a hole undergo momentum changes within their own subbands;
- Thirdly, we assume that the potentiometric contacts are connected to the electron subsystem. As illustrated in Figure 1d, the gate covers the sample, but the contacts are only partially covered. This implies that the Fermi energy in the areas not under the gate corresponds to a gate voltage of zero, which, for all three samples, corresponds to the electron band scenario. Since the contacts contain only electrons, and given that at low temperatures, the electron and hole subsystems do not interact, the potentiometric contacts provide potentials of exclusively electron subsystems. Furthermore, since the current contacts have a larger area than the potentiometric contacts (see Figure 1d), we can also assume that the potentials of the subsystems would be balanced, allowing the current to be carried by both electrons and holes.

All of these considerations lead us to a qualitative understanding of the transport in the system. At low temperatures, the electron network and the sea of holes (see Figure 7) interact weakly, allowing the potentials of the subsystems to differ. Since the potentiometric contacts are connected to the electron subsystem, while the current flows through both, we can treat the sea of holes as a shunt resistance. Therefore, the measured conductivity at the lowest temperature (see Figure 2a) with significant fluctuations represents the conductivity of the electron network, which is renormalized by the shunting conductivity of the holes. To estimate the ratio of their conductivities, we take the mobilities ratio $\mu_{e}/\mu_{h}\approx 10$ and the concentrations ratio $N_{s}/P_{s}\approx 10^{-2}$ (see Figure 1), which results into the one order difference. Hence, the actual fluctuation amplitude of the electron network’s conductivity, according to our considerations, is approximately one order of magnitude lower than what is shown in Figure 2c. Thus, the maximum value of the fluctuations amplitude would be around $e^{2}/h$, which appears to be more plausible than the measured 10 $e^{2}/h$ (see Figure 2c).

Then, we need to describe the magnetic field dependences of the fluctuations from Figure 2c and inset in Figure 6b. Since the measured fluctuating conductivity at low temperatures is considered to be just a rescaled conductivity of the electron percolation network, the magnetic field dependences of the fluctuations amplitude have to be described only by the properties of the electron percolation network. Firstly, at zero magnetic field, we observe some fluctuations, but they are relatively weak. The gate voltage variation changes the average concentration of the electrons, thus changing the picture of the percolation ways in the network by filling or emptying different connections. Secondly, with the magnetic field *B*, all the different closed electron circuits (or blobs) receive an additional phase of eBS/ℏ, where *S* is the area of the blob, which was estimated as 0.04 μm${}^{2}$ from the oscillations-like behavior from Figure 4. When the phase shift is comparable to π, all the resistances of the blobs become very sensitive to any variations of the network’s geometry, and thus, the fluctuations’ amplitude dramatically increases. This happens at the magnetic fields around 50 mT, that agrees with the experiment where the amplitude at this field is already large. Thirdly, at the fields higher than 0.5 T, the fluctuations vanish. This aspect cannot be explained by our independent electron percolation network model alone. One possible reason for this shutdown is the transition of the hole subsystem to the Shubnikov–de Haas regime, which may somehow disrupt the percolation network. However, this idea should be supported by a specific mechanism within a microscopic theory that accounts for the contribution of holes to the formation of the electron percolation network.

Finally, we come to the temperature dependence. At higher temperatures, electron-hole scattering occurs, resulting in the intermixing of the electron and hole subsystem potentials. Given that the hole liquid continues to be the primary conductor of the system, the fluctuating potential of the electron network bounds to the classical potential of the holes, consequently causing a reduction in the amplitude of the fluctuations and simultaneously preserving the average conductivity. This consideration is supported by the temperature dependence of the fluctuations’ amplitude from Figure 3b and the inset in Figure 6a, where, at low temperatures, the amplitude saturates to a finite value, but at higher temperatures, it exponentially decreases. Apart from the intermixing of the electron and hole subsystems, the phase decoherence length also shortens with the temperature increase due to the inelastic scattering, which can be a second possible mechanism of the fluctuation shutdown.

## 5. Conclusions

In conclusion, this work reports the first observation and study of unusual mesoscopic conductance fluctuations in HgTe-based two-dimensional semimetal in a weak localization regime. These fluctuations exist in macroscopic samples with characteristic sizes higher than 100 μm. They have an anomalously high amplitude (≈10 $e^{2}/h$) and anomalous gate voltage, magnetic field (quasiperiodicity and suppression at B≈ 0.5 T), and temperature (exponentially strong) dependences. Also, their amplitude critically depends on the degree of disorder. Moreover, it was found that at the same conductivity value (about 1 kΩ/☐), they are completely absent in the electron metal state realized in the same sample.

We propose a qualitative model of an electronic percolation network that explains why mesoscopic conductance fluctuations are possible to observe in macroscopic samples and why they could be that large. Nevertheless, some aspects of the theory require further theoretical validation. Since the theory mostly provides qualitative results, the development of a comprehensive theory that both offers a microscopic description of such a percolation network’s formation and explains all the main experimental properties of discovered mesoscopic conductance fluctuations is an intriguing challenge for the theory of two-dimensional correlated systems.

## Data Availability

The data that support the findings of this study are available from the corresponding author upon reasonable request.

## Abbreviations

The following abbreviations are used in this manuscript:

UCF	Universal conductance fluctuations
QW	Quantum well
